# A primary tumor gene expression signature identifies a crucial role played by tumor stroma myofibroblasts in lymph node involvement in oral squamous cell carcinoma

**DOI:** 10.18632/oncotarget.20645

**Published:** 2017-09-05

**Authors:** Gianluigi Mazzoccoli, Stefano Castellana, Massimo Carella, Orazio Palumbo, Cristiana Tiberio, Caterina Fusilli, Daniele Capocefalo, Tommaso Biagini, Tommaso Mazza, Lorenzo Lo Muzio

**Affiliations:** ^1^ Department of Medical Sciences, Division of Internal Medicine and Chronobiology Unit, IRCCS Scientific Institute and Regional General Hospital “Casa Sollievo della Sofferenza”, S. Giovanni Rotondo (FG), Italy; ^2^ Bioinformatics Unit, IRCCS Scientific Institute and Regional General Hospital “Casa Sollievo della Sofferenza”, S. Giovanni Rotondo (FG), Italy; ^3^ Medical Genetics, IRCCS Scientific Institute and Regional General Hospital “Casa Sollievo della Sofferenza”, S. Giovanni Rotondo (FG), Italy; ^4^ Department of Clinical and Experimental Medicine, University of Foggia, Foggia, Italy

**Keywords:** OSCC, gene signature, metastasis, transcriptome, myofibroblast

## Abstract

Oral squamous cell carcinoma (OSCC) is the most common oral and pharyngeal cancer, and is responsible of approximately 3% of cancers in men and 2% in women in the Western World, with increasing incidence rates in developing countries. Early detection by screening is necessary to prevent fatal disease because early, curable lesions are rarely symptomatic. The overall 5-yr survival rate is approximately 50% when surgery, radiation, or both are employed as treatment options, but lymph node involvement greatly influences this estimate, by decreasing the survival rate by about 50%. Here, we aimed at finding genetic signatures associated with lymph node metastasis in OSCC patients. We addressed this issue by whole transcriptome analysis through microarray expression profiling of a set of OSSC specimens of patients without lymph node involvement (10 patients, mean age ± SD 61.2±13.8, male 7, female 3) and with lymph node involvement (11 patients, mean age ± SD 62.1±15.1, male 8, female 3). We evidenced a gene expression signature associated to muscle contraction-related genes in specimens obtained from OSCC patients with lymph node involvement. This gene signature suggests the presence of myofibroblasts in tumor stoma of patients with lymph node involvement and emphasizes the decisive role played by myofibroblasts probably through their secretome in determining OSCC invasiveness.

## INTRODUCTION

Oral squamous cell carcinoma (OSCC) represents more than 95% of the carcinomas of the oral cavity and the most common form of head and neck cancer, corresponding to the sixth commonest cancer in males, the tenth commonest site of cancer in females, and a major health problem as well as a leading cause of death in developing countries [1]. In spite of the progress in diagnosis and treatment of other malignant tumors, OSCC is characterized by dreadfully insufficient therapeutic approaches and meager rates of response to treatment that lead to limited 5-year survival rate not exceeding 55%, mainly caused by locally aggressive tumor phenotypes, and the survival rate diminishes by about 50% in the presence of lymph node involvement [2, 3]. Tumor biological behavior or patients’ outcome is not adequately predicted by clinic-pathological characteristics and staging systems taking into account anatomical parameters, for example the Tumor-Nodes-Metastasis (TNM) classification. A crucial role in the anatomical changes, molecular alterations and pathway derangements that support the deregulation of physiological processes causing onset and progression of malignant neoplasms is played by the tumor microenvironment [4–7]. In particular, cancer related death is caused principally by metastatic progression, and the interplay between neoplastic lesions and host tissue, in addition to the mutual influence among cancer cells and the niche created by the microenvironment encompassing the tumor-associated stroma, drives the progression of cancer from onset to metastatization [8]. The process of tumor progression is driven by chemo/cytokines and growth factors produced through autocrine or paracrine secretion by elements of the cancer tissue and tumor microenvironment exerting their effects on neighboring cells [9]. Importantly, in the framework of malignant neoplasms the same factor may act as a tumor suppressor if greatly expressed in tumor tissue, or as an oncogene if greatly expressed in the tumor-associated stroma [10]. In this way, the microenvironment enclosing and sustaining the tumor tissue can impact malignant behavior of a developing neoplastic lesion and lead to metastatic dissemination[11]. This interplay may impact significantly OSCC onset and progression, as well as patients’ outcome and response to therapy, and there is urgent need for a better knowledge of pathophysiological mechanisms and molecular events involved in oral squamous cell carcinogenesis to advance prognostic stratification and clinical management of OSCC patients.

A number of candidate genes involved in apoptosis, cell cycle and cell proliferation have been found associated with OSCC progression [12–15]. Anyway, it is not possible to predict the biological behavior of OSCC on the basis of the differential expression of a single gene. Transcriptome-wide analysis performed by means of high-throughput gene expression profiling techniques permits the evaluation of expression levels of thousands of genes and allows to identify gene expression signatures to screen patient subsets and stratify prognostic classes [16, 17]. Besides, better understanding of the processes involved in carcinogenesis and metastatization was afforded by gene expression profiling using microarray hybridization, which rather than pinpointing the expression of a small number of genes makes available genomic-scale expression outlines, consenting the investigation of genetic expression variability in the background of broader genetic contexts and identification of specific biochemical pathways that might be targeted by new therapeutic agents. A number of studies have addressed this issue in OSCC patients and identified gene signatures comprising functional gene groups correlated with aggressiveness and invasiveness. The data allow patient subsets clustering and corroborate the evidence that primary OSCC with and without metastatic lymph node dissemination are hallmarked by measurable differences in gene expression, able to predict the node status and overall prognosis of an independent set of patients based on the gene expression of the primary tumor [18–23]. Anyway, none of these results highlighted the role of tumor microenvironment, implying the need for further studies pinpointing processes in both the tumor and nontumor cells.

We planned to evaluate a series of OSCC specimens obtained from patients with and without lymph node involvement by means of gene expression microarray technology and both a qualitative and a quantitative enrichment analysis. Our analyses identified a significant enrichment of muscle contraction-related genes on the side of lymph node-positive OSCC, in respect to specimens obtained from lymph node-negative patients, suggesting a crucial role played by myofibroblasts present in tumor stroma in driving invasive behavior.

## RESULTS

**Figure 1:**
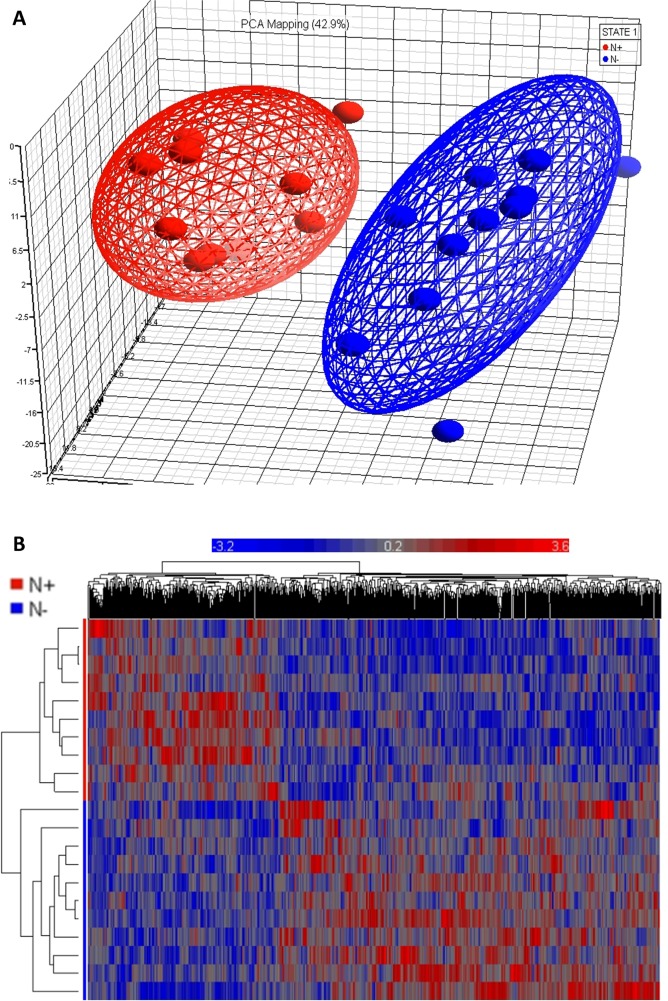
Microarray data analysis **(A)** Principal Component Analysis (PCA) plot shows two distinct clusters: patients with oral squamous cell carcinoma (OSCC) with and without lymph node involvement (N+ and N-, as outlined with red and blue balls and lines, respectively). **(B)** Clustered heat map of differentially expressed genes in the primary tumor of OSCC patients with and without lymph node involvement demonstrates that the genes are clustered closely for N+ and N- patients. Horizontal axis shows genes used for clustering and vertical axis shows sample clustering.

The 21 individuals under examination, with and without lymph node involvement, clustered in an unsupervised fashion by their gene expression profiles, as from the PCA and heatmap plots in Figure 1. A total of 41 genes were differentially expressed when fixing the fold change barrier to ±2 at a nominally significant p-value (p < 0.05) and remained significant after correcting for multiple testing (FDR < 0.05): 19 genes were expressed at higher levels and 22 gene were expressed at a lower level in tumor specimens from OSCC patients with lymph node involvement (Table 1). Differentially regulated genes in the primary tumor of patients with OSCC with and without lymph node involvement are rendered as heat map and PCA in Figure 1. The most striking functional difference between samples obtained from lymph node-negative and lymph node-positive patients consisted in the significant enrichment of muscle contraction-related genes in favor of lymph node-positive patients. *Muscle contraction* (GO:0006936, enrichment ratio = 12.62, adjusted p-value = 1.94e-05) resulted to be the most significant *Biological Process*, according to WebGestalt analysis. CHRNA1, CKMT2, DSC2, MYBPC2, MYOT, NEB, SMPX, and TNNC1 genes represented this category, and were found to be highly expressed in lymph node-positive patients. Other significant categories, indirectly associated to *muscle contraction*, were: *contractile fiber* (GO:0043292, adjusted p-value = 2.66e-06); *sarcomere* (GO:0030017, adjusted p-value = 1.04e-05); *myofibril* (GO:0030016, adjusted p-value = P=2.13e-05). Most of these categories turned out to be enriched by up-regulated genes in specimens from lymph node-positive patients only (adjusted p-value for *muscle contraction*: 4.05e-07; *myofibril* cellular component: 1.03e-07; *sarcomere* cellular component: 4.89e-08), and not by up-regulated genes in specimens from lymph node-negative patients, thereby indicating that they characterize exclusively the lymph node-positive sub-set of patients. DAVID confirmed these results, by ranking a cluster containing *muscle contraction* process with the highest value (enrichment score = 3.12, GO:0006936, FDR=0,04). Still, highly expressed genes in specimens from lymph node-negative patients were not assigned to any significant functional cluster. Similar results were achieved even if lowering the fold change barrier to ±1.5; DMD, MURC, MYL3, MYLPF, MYOT, MYOZ2, NEB, RYR1, SYNM, TNNC1, TNNI1, TNNI2, TPM2, TPM3, and TRIM54 represented significantly the abovementioned ontological terms and were almost exclusively expressed in specimens from lymph node-positive patients.

**Table 1:** List of differentially regulated genes In the primary tumor of OSCC patients with and without lymph node involvement

Official Symbol	Official Full Name	p-value	Fold-Change
Differentially expressed genes that increase in the primary tumor of patients with OSCC with lymph node involvement			
*CHRNA1*	cholinergic receptor, nicotinic, alpha 1 (muscle)	0.03482	2.00
*MYLPF*	myosin light chain, phosphorylatable, fast skeletal muscle	0.03251	2.04
*ERV3-1*	endogenous retrovirus group 3, member 1	0.0038	2.08
*ERAP2*	endoplasmic reticulum aminopeptidase 2	0.04023	2.10
*SNHG1*	small nucleolar RNA host gene 1 (non-protein coding)	0.01481	2.13
*ENO3*	enolase 3 (beta, muscle)	0.01708	2.14
*MURC*	muscle-related coiled-coil protein	0.03014	2.25
*CKMT2*	creatine kinase, mitochondrial 2 (sarcomeric)	0.01366	2.26
*LMOD3*	leiomodin 3 (fetal)	0.03237	2.26
*CHI3L2*	chitinase 3-like 2	0.01282	2.32
*STAC3*	SH3 and cysteine rich domain 3	0.03348	2.32
*SERPINA3*	serpin peptidase inhibitor, clade A (alpha-1 antiproteinase, an	0.04853	2.39
*CMYA5*	cardiomyopathy associated 5	0.04278	2.43
*LRAT*	lecithin retinol acyltransferase (phosphatidylcholine--retinol O-ac	0.04715	2.50
*SMPX*	small muscle protein, X-linked	0.03998	2.57
*MYBPC2*	myosin binding protein C, fast	0.00594	2.91
*TNNC1*	troponin C type 1 (slow)	0.04493	2.95
*MYOT*	myotilin	0.03909	2.99
*NEB*	nebulin	0.04540	3.45
Differentially expressed genes that decrease in the primary tumor of patients with OSCC with lymph node involvement			
*KRT17*	keratin 17	0.03647	−2.00
*ENTPD3*	ectonucleoside triphosphate diphosphohydrolase 3	1.3e-005	−2.04
*SLC2A1*	solute carrier family 2 (facilitated glucose transporter), member	0.00615	−2.06
*NQO1*	NAD(P)H dehydrogenase, quinone 1	0.03367	−2.07
*ALDH3A1*	aldehyde dehydrogenase 3 family, member A1	0.03221	−2.08
*SOX2*	SRY (sex determining region Y)-box 2	0.04546	−2.11
*PPP2R2C*	protein phosphatase 2, regulatory subunit B, gamma	0.02403	−2.13
*NLGN4X*	neuroligin 4, X-linked	0.0035	−2.25
*SERPINB11*	serpin peptidase inhibitor, clade B (ovalbumin), member 11 (ge	0.02070	−2.30
*IGFBP2*	insulin-like growth factor binding protein 2, 36kDa	0.00632	−2.32
*DSC2*	desmocollin 2	0.04781	−2.33
*DAPL1*	death associated protein-like 1	0.01156	−2.51
*PTHLH*	parathyroid hormone-like hormone	0.00388	−2.53
*SLC7A11*	solute carrier family 7 (anionic amino acid transporter light ch	0.03223	−2.72
*GJB6*	gap junction protein, beta 6, 30kDa	0.01592	−2.77
*LOC344887*	NmrA-like family domain containing 1 pseudogene	0.04576	−2.79
*SLC7A8*	solute carrier family 7 (amino acid transporter light chain, L sy	7.7e-005	−2.81
*EPGN*	epithelial mitogen homolog (mouse)	0.02931	−3.35
*ADH7*	alcohol dehydrogenase 7 (class IV), mu or sigma polypeptide	0.03912	−3.36
*SPINK6*	serine peptidase inhibitor, Kazal type 6	0.00436	−3.53
*MMP10*	matrix metallopeptidase 10 (stromelysin 2)	0.00940	−4.23
*NEFL*	neurofilament, light polypeptide	0.0001	−4.26

OSCC= oral squamous cell carcinoma; up-regulated and down-regulated genes between OSCC specimens obtained from lymph node positive versus lymph node negative patients. Selection criteria were p < 0.05 (based on ANOVA test) and a fold-change barrier of ±2.

WebGestalt and GeneCodis3 agreed on the enrichment of a *myocyte enhancer factor recognition site*, recurrent in five genes of our dataset (adjusted p-value = 0.0007): the HMEF2_Q6 binding site. Four out of these genes (CKMT2, SMPX, SOX2 and TNNC1) were associated to the GO terms: *muscle contraction*, *sarcomere* and *myofibril*, and were highly expressed in samples from lymph node positive patients. Only one gene, ENO3, was associated to the *HMEF2_Q6* binding site, still being down regulated in the lymph node positive patients’ dataset. Significant co-occurrences of HMEF2_Q6 sites in four of the previously mentioned genes (CKMT2, ENO3, SMPX and TNNC1) were also confirmed by GeneCodis3 (adjusted p-value = 2.26e-05).

Most of the “muscle tissue-related” genes (CKMT2, MYOT, NEB, SMPX, TNNC1) generally exhibited elevate expression levels in oral mucosa, as resumed by GENEVESTIGATOR 4.0 software. On the contrary, the same set of genes exhibited a generally low-level expression in OSCC cell lines (SCC-4, SCC-9, SCC-15, SCC-25, BHY, BICR1 and HN)(Ncbi GEO ID: GSE36133 or Ebi Array Express ID: E-GEOD-36133). Furthermore, TNNC, CKMT2 and NEB1 turned out to be slightly under-express in head and neck squamous cell carcinoma with respect to normal tissue (E-GEOD-6631; log2FC=-1.3, −1.3 and −1, respectively, p-values <0.05).

The expression of muscle tissue associated genes in samples from lymph node positive patients but not in OSCC cell lines suggests that such gene signature may derive from myofibroblasts, which are absent in normal oral mucosa and constitute the OSCC stroma [29].

We also studied the expression profile of the 22 genes over-expressed in lymph node negative patients’ samples. We noticed that two relevant genes: DSC2 (encoding desmocollin 2, involved in cell adhesion, GO:0007155), and KRT17 (encoding keratin 17, involved in morphogenesis of an epithelium, GO:0002009; signal transduction GO:0007165; epidermis development, GO:0008544; positive regulation of cell growth, GO:0030307) are highly expressed in oral squamous cell carcinoma cell line, while they are down regulated in specimens from patients with lymph node involvement. KRT17 expression level is confirmed to be elevate in most OSCC cell lines (BHY, BICR 1, BICR 7, HN, as evidenced in RNAseq (ArrayExpress ID: “E-MTAB-2706”) and array public experiments (E-GEOD-30784, log2FC=4.1 for “oral squamous cell carcinoma vs normal samples”, p-value=2.19E^−38^). Interestingly, under-expression of KRT17 gene within OSCC samples without lymph node involvement was previously reported [30].

Interestingly, also the NEFL gene (encoding the light chain neurofilament protein and involved in cell death, GO:0008219; intermediate filament organization, GO:0045109) was found to be under-expressed in OSCC specimens with lymph node involvement: it seems congruent with its role in the promotion of apoptosis and prevention of cancer invasion, as previously reported in head and neck squamous cell carcinoma cell lines [31]. Among the other, down-regulated genes in samples obtained from lymph node positive patients, we found that IGFBP2 (encoding insulin-like growth factor binding protein 2 and involved in regulation of cell growth, GO:0001558; regulation of insulin-like growth factor receptor signaling pathway, GO:0043567) and PTHLH (encoding parathyroid hormone-like hormone and involved in cell-cell signaling, GO:0007267; endoderm development, GO:0007492; positive regulation of cell proliferation, GO:0008284; negative regulation of cell proliferation, GO:0008285; epithelial cell differentiation, GO:0030855) are generally down-regulated also in OSCC cell line and in OSCC metastasis [18].

Other interesting genes found down-regulated in the specimens obtained from patients with lymph node involvement are: DAPL1 (encoding death-associated protein like 1 and involved in apoptotic process, GO:0006915; cell differentiation, GO:0030154), EPGN (encoding epithelial mitogen and involved in activation of MAPK activity; GO:0000187; angiogenesis, GO:0001525; positive regulation of cell proliferation, GO:0008284; positive regulation of epidermal growth factor-activated receptor activity; GO:0045741; positive regulation of mitosis, GO:0045840; positive regulation of epithelial cell proliferation, GO:0050679), GJB6 (encoding gap junction protein, beta 6, 30kDa, and involved in apoptotic process, GO:0006915; cell communication, GO:0007154; negative regulation of cell proliferation, GO:0008285), NQO1 (encoding NAD(P)H dehydrogenase, quinone 1 and involved in regulation of cellular amino acid metabolic process, GO:0006521; superoxide metabolic process, GO:0006801; xenobiotic metabolic process, GO:0006805; response to oxidative stress, GO:0006979). Most of these genes exhibited different expression levels in oral mucosa and OSCC cell lines (SCC-4, SCC-9, SCC-15, SCC-25, BHY, BICR1 and HN) as evidenced by data extracted by Genevestigator 4.0, selecting “oral mucosa” and “OSCC” as sample tissues (raw data from GEO Ser). DAPL1 is generally under-expressed in OSCC samples with respect to normal tissue from controls (E-GEOD-30784, log2FC=-2.3, p-value=2.39E^−9^) and NQO1 appears to have elevated baseline expression levels in BHY, BICR1 and HN OSCC lines (E-MTAB-2706).

**Figure 2:**
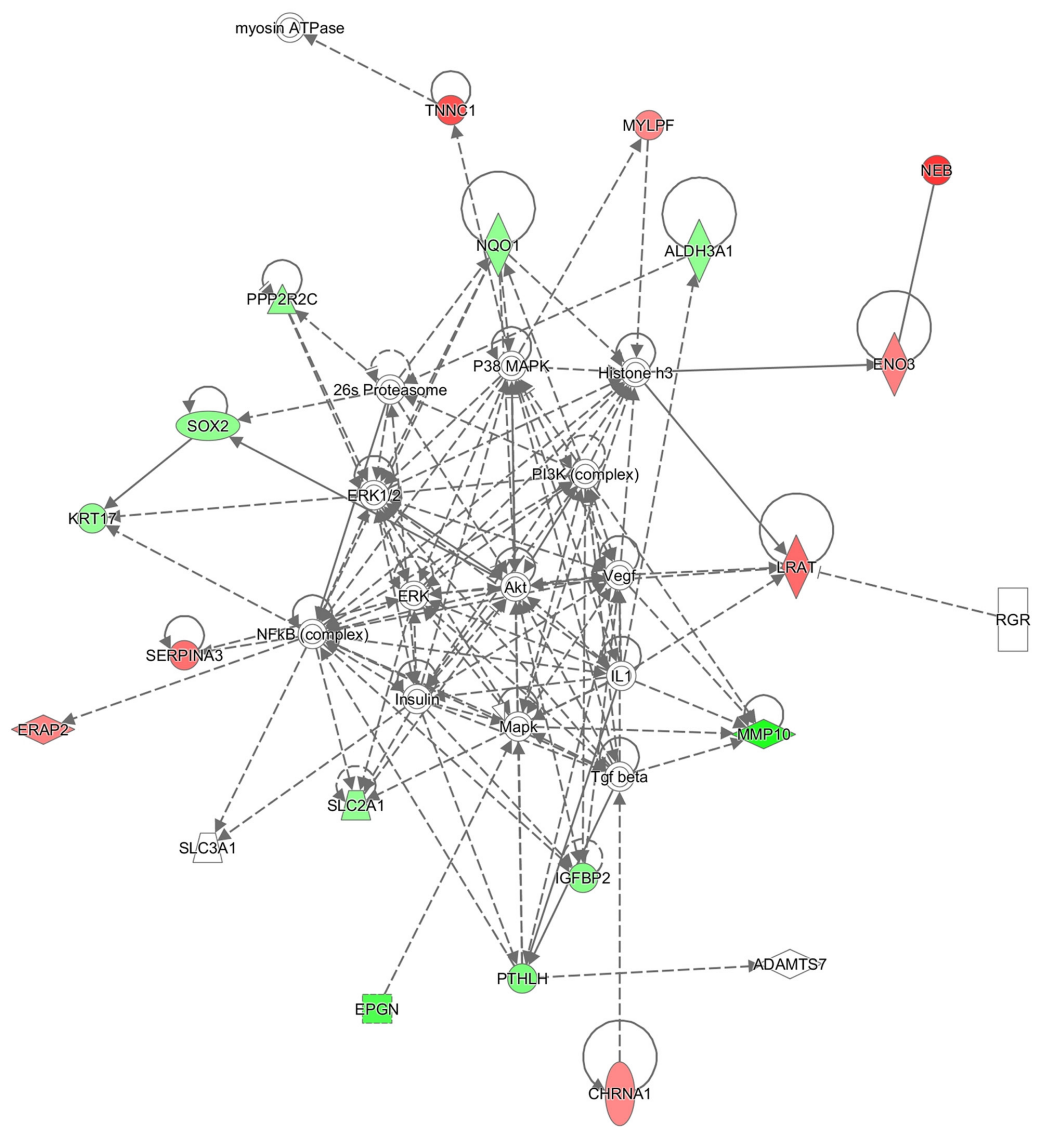
Ingenuity pathway analysis Embryonic Development, Organ Development, Organ Morphology network illustrated as a graph according to differentially expressed genes. A node represents a differentially expressed gene. The node color is associated with expression level. Red indicates that the gene is down-regulated while green indicates that the gene is up-regulated. Grey indicates that the gene expression level is unpredicted.

**Figure 3:**
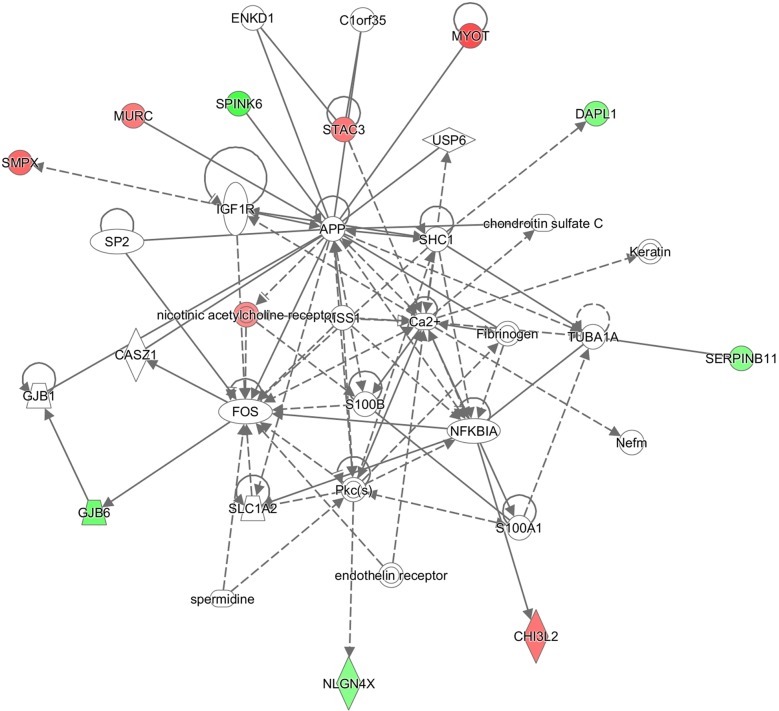
IPA enrichment analysis Cell Death and Survival, Skeletal and Muscular Disorders, Neurological Disease network illustrated as a graph according to differentially expressed genes. A node represents a differentially expressed gene. The node color is associated with expression level. Red indicates that the gene is down-regulated while green indicates that the gene is up-regulated. Grey indicates that the gene expression level is unpredicted.

**Figure 4:**
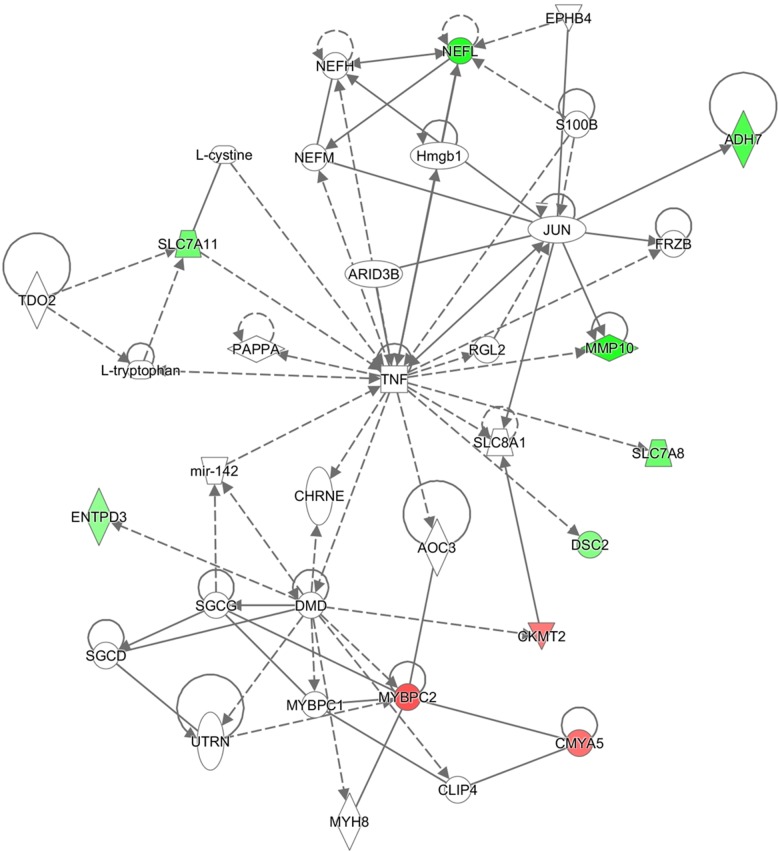
Ingenuity pathway analysis Cellular Assembly and Organization, Nervous System Development and Function network illustrated as a graph according to differentially expressed genes. A node represents a differentially expressed gene. The node color is associated with expression level. Red indicates that the gene is down-regulated while green indicates that the gene is up-regulated. Grey indicates that the gene expression level is unpredicted.

IPA sorted out the canonical pathway Embryonic Stem Cell Differentiation into Cardiac Lineages with -log(p-value) 1.75E00, the function Muscle Contraction (p-value 8,66E-11, enriched by CHRNA1, CKMT2, MYBPC2, MYLPF, MYOT, NEB, PTHLH, SMPX, STAC3, TNNC1), and three interesting networks:

1. Embryonic Development, Organ Development, Organ Morphology (enriched by 26s Proteasome, ADAMTS7, Akt, ALDH3A1, CHRNA1, ENO3, EPGN, ERAP2, ERK, ERK1/2, Histone h3, IGFBP2, IL1, Insulin, KRT17, LRAT, Mapk, MMP10, MYLPF, myosin ATPase, NEB, NFkB (complex), NQO1, P38 MAPK, PI3K (complex), PPP2R2C, PTHLH, RGR, SERPINA3, SLC2A1, SLC3A1, SOX2, Tgf beta, TNNC1, Vegf) (Figure 2);
2. Cell Death and Survival, Skeletal and Muscular Disorders, Neurological Disease (enriched by APP, C1orf35, Ca2+, CASZ1, CHI3L2, chondroitin sulfate C, DAPL1, endothelin receptor, ENKD1, Fibrinogen, FOS, GJB1, GJB6, IGF1R, Keratin, KISS1, MURC, MYOT, Nefm, NFKBIA, nicotinic acetylcholine receptor, NLGN4X, Pkc(s), S100A1, S100B, SERPINB11, SHC1, SLC1A2, SMPX, SP2, spermidine, SPINK6, STAC3, TUBA1A, USP6) (Figure 3);
3. Cellular Assembly and Organization, Nervous System Development and Function, Hereditary (enriched by ADH7, AOC3, ARID3B, CHRNE, CKMT2, CLIP4, CMYA5, DMD, DSC2, ENTPD3, EPHB4, FRZB, Hmgb1, JUN, L-cystine, L-tryptophan, mir-142, MMP10, MYBPC1, MYBPC2, MYH8, NEFH, NEFL, NEFM, PAPPA, RGL2, S100B, SGCD, SGCG, SLC7A8, SLC7A11, SLC8A1, TDO2, TNF, UTRN) (Figure 4).

## DISCUSSION

The numerous constituents of the tumor stroma participate in different ways realizing a complex system that decisively impinges on cancer behavior and disease outcome, but on the other hand could represent targets for therapeutic approaches [32]. In this regard, an important role is played by cancer associated fibroblasts, found in the nearness in the developing and advancing cancer and able to promote tumor development and metastasis through secretion of chemokines. A bidirectional cross-talk between tumor cells and cancer associated fibroblasts drives discharge by cancer cells of factors augmenting the ability of the fibroblasts to secrete an assortment of tumor-promoting chemokines, which subsequently impact on the malignant behavior of tumor cells upholding their proliferation, migration, and invasiveness [33]. The chemokines secreted by cancer associated fibroblasts impinge on the tumor microenvironment as well, stimulating neoangiogenesis and in some instances an important presence of cancer-supporting macrophages in tumors [34]. Tumorigenicity is propped up by a number of proteins secreted by cancer associated fibroblasts and, in turn, factors secreted by cancer cells induce secretion of these proteins, exerting considerable effects on the ability of fibroblasts to promote tumor growth [35, 36]. This interplay seems to play a crucial role in OSCC where the crosstalk between carcinoma associated fibroblasts and OSCC cells influences phenotype transition of cancer cells as a prerequisite for tumor progression, conditionating patients’ outcome and therapies efficacy, and activated fibroblasts and in particular myobroblasts are often found in the stroma of OSCC, representing an important risk factor of patient's shortened survival [37–39]. Myofibroblasts in the stroma of OSCC may influence proliferation and invasion, resulting in more aggressive tumor, and tumor-associated myofibroblasts produce higher levels of growth factors compared to tumor-associated fibroblasts, and induce *in vitro* increased production of matrix metalloproteinases (MMP) accompanying invasion of OSCC cells [38]. In agreement with these evidences we found a significant enrichment of muscle contraction-related genes in the specimens obtained from lymph node-positive patients, and in particular this category was enriched by the genes TNNC1, CKMT2, SMPX, MYOT, CHRNA1, MYBPC2, NEB, found upregulated, and DSC2, found downregulated.

Similarly, other categories found enriched by up-regulated genes in specimens from lymph node-positive patients only were *contractile fiber*, *sarcomere*, *myofibril*, which are in relationship with the immunoistochemical evidence of myofibroblast occurrence in tumor stoma of node positive patients and support the prominent role for myofibroblasts in OSCC invasiveness [40, 41]. In addition, bioinformatics tools for functional genomic, proteomic and large-scale genetic studies agreed on the enrichment of a *myocyte enhancer factor recognition site*, the *HMEF2_Q6* binding site, recurrent in five genes of our dataset. Four out of these genes (TNNC1, CKMT2, SMPX and SOX2) were associated to the GO terms: *muscle contraction*, *sarcomere* and *myofibril*, and were highly expressed in samples from lymph node positive patients. The most important activated stromal fibroblast phenotype is the myofibroblast, and the presence of myofibroblasts in tumour stroma is notably associated with a poor prognosis of OSCC patients [39]. Among the up-regulated genes we did not find ACTA-2, encoding α-smooth muscle actin (α-SMA), the most commonly used myofibroblast molecular marker, probably in relation to the differentiation process. Transdifferentiation of myofibroblasts is induced in the course of oral carcinogenesis and in particular in the invasive stage of OSCC irrespective of the epithelial cell differentiation. The protomyofibroblast, whose stress fibers contain only β- and γ-cytoplasmic actins, is a stable phenotype *in vitro*, but is an intermediate step in most *in vivo* conditions where it may progress, but not necessarily always, into the appearance of the differentiated myofibroblast, which is characterized by *de novo* expression of α-SMA [42].

Rearding DSC2, this gene encodes desmocollin 2, a calcium-dependent glycoprotein member of the cadherin superfamily, which in epithelial cells makes up the adhesive proteins of the desmosome cell-cell junction and is required for cell adhesion and desmosome formation. The down-regulated expression of DSC2 in specimens from patients with lymph node involvement in concert with the down-regulation of KRT17, encoding keratin 17, which are normally greatly expressed in healthy mucosa, represent a further hallmark of invasive OSCC. Regarding to SOX2, a putative cancer stem-like cell/cancer-initiating cell marker for several human malignancies, it was found down-regulated in the specimens obtained from lymph node positive OSCC. In previous studies its immunohistochemical staining has been correlated with poor overall, cancer-specific and disease-free survivals in patients with histologically node-negative tongue localized OSCC [43] and with lymph node metastasis in OSCC when the immunohistochemistry analysis evidenced a diffuse staining pattern [44]. Among the genes found down-regulated in the specimens obtained from lymph node positive patients, the NQO1 gene encodes the phase II drug-metabolizing enzyme NAD(P)H:quinine oxidoreductase 1 (NQO1) and is considered significant for susceptibility to general carcinogenesis, and the genomic mutation in NQO1 is frequently found in cancer of colon, bladder, lung and pediatric leukemia. In addition, the genomic polymorphism in NQO1 is related to OSCC susceptibility, representing a genetic risk factor for OSCC carcinogenesis [45]. Also EPGN was found down-regulated in the specimens obtained from lymph node positive patients, and interestingly this gene was found upregulated in relapsed or metastatic head and neck squamous cell cancer patients treated with first-line cetuximab and platinum therapy who presented long progression-free survival [46]. In OSCC specimens obtained from lymph node positive subjects we found downregulation of SERPINB11 and MMP10, and our data are in agreement with reports showing down-regulated expression of SERPIN genes located on chromosome 18q21 in OSCC [47], and the reliability of MMPs as molecular markers for monitoring progression from normal tissue to dysplasia and OSCC more than to invasive behavior of tumor cells [48].

On the other hand, in lymph node positive OSCC patients we found upregulation of SERPINA3, the gene encoding the protease inhibitor alpha 1-antichymotrypsin. This finding is in agreement with the association of SERPINA3 up-regulation with poorer survival in patients with HLA-positive cervical carcinoma [49], and with disseminated disease in patients affected by colorectal adenocarcinoma [50].

In conclusion, we performed a transcriptome-wide analysis on specimens obtained from lymph node-positive and lymph node-negative OSCC patients by means of high-throughput gene expression profiling techniques. A qualitative and quantitative enrichment analysis evidenced a gene expression signature associated with spread of cancer to cervical lymph nodes. This category is related to muscle contraction-related genes, suggesting the presence of myofibroblasts in tumor stroma of patients with lymph node metastasis. This gene signature found in the specimens obtained from lymph node-positive patients corroborates results of immunoistochemical studies performed in OSCC patients evidencing positivity of myofibroblast markers associated to invasiveness [51–54]. This evidence underscores the crucial role played by myofibroblasts probably through their secretome in determining OSCC invasiveness. In addition, this gene signature corroborates the reliability of myofibroblastic stroma to predict OSCC mortality and the importance of molecular classification to sort patients with aggressive cancers, when used for prognostic application in alternative or in association to disease staging through TNM system.

## MATERIALS AND METHODS

### Patients

Tumor tissue specimens were collected from 21 patients (mean age ± SD 61.56 ±13.95 yrs, age range 47-75 yrs, male 15, female 6), affected by OSCC during surgical intervention, 10 patients without lymph node involvement (mean age ± SD 61.2±13.8 yrs, age range 47-72 yrs, male 7, female 3) and 11 patients with lymph node involvement (mean age ± SD 62.1±15.1 yrs, age range 48-75 yrs, male 8, female 3). Written informed consent to a protocol reviewed and approved by the Institutional Ethical Committee was obtained from patients to use their surgical specimens for research purposes. Criteria of inclusion were: diagnosis of OSCC, no previous radiotherapy, no previous chemotherapy; criteria of exclusion were: previous treatments, infective diseases or salivary gland diseases. Primary treatment was based on surgery and local resection was governed by T categories. Part of each sample was frozen in liquid nitrogen and stored at −80°C until RNA extraction, and the remaining part was used for histopathological analysis. Patients’ characteristics are described in Table 2.

**Table 2:** Clinical and pathological features of oral squamous cell carcinoma patients

	Total	N-	N+
Number	21	10	11
Age (Mean ± SD)	61.56 ± 13.95	61.20 ± 13.79	62.00 ± 15.10
Gender			
- Male	15	7	8
- Female	6	3	3
Tumour location			
- Tongue	5	2	3
- Lip	2	0	2
- palate	3	2	1
- gingival	4	3	1
- floor	2	1	1
- mandible	4	1	3
- buccal mucosa	1	1	0
Histologic Type			
- G1	2	2	0
- G2	10	5	5
- G3	9	3	6
Depth of tumour invasion			
- T1	4	2	2
- T2	10	6	4
- T3	3	0	3
- T4	4	2	2
Lymph node involvement			
- Nx	3	0	3
- N0	10	10	0
- N1	4	0	4
- N2	4	0	4
Metastasis			
- Mx	10	4	6
- M0	11	6	5
- M1	0	0	0
TNM STAGE			
- I-II	13	8	5
- III-IV	8	2	6
Grading			
- G1-G2	12	7	5
- G3-G4	9	3	6
Neural invasion			
- No	9	4	5
- Yes	12	6	6

### RNA extraction from fresh frozen tissue and first-strand cDNA synthesis

About 150 to 200 mg of fresh frozen tissues were sampled, total RNA was obtained by phenol extraction (TRIzol Reagent, Invitrogen Corporation, Carlsbad, CA, USA), and subsequently purified by column chromatography (RNeasy Mini Kit, Qiagen, Valencia, CA, USA). The amount of extracted RNA was checked by Nano Drop Spectrophotometer whereas RNA integrity was monitored using Agilent 2100 Bioanalyzer after subsequent digestion by DNaseI. Next, 1.0 mg of total RNA was reversed transcribed using the High-Capacity cDNA Archive Kit following the manufacturer's instructions (Applied Biosystems, Applera, Foster City, CA, USA).

### DNA microarray assays

Whole genome gene expression profiling experiments was performed by using Affymetrix GeneChip1 Human Gene 1.0 ST Arrays. Samples were prepared for Affymetrix GeneChip1 Human Gene 1.0 ST Array System that interrogate 28,869 well annotated genes by using an average of 26 probes per gene; 100 ng of total RNA were processed according to the GeneChip Whole Transcript (WT) Sense Target Labeling Assay as provided by the manufacturer (Affymetrix). A random priming method was used to generate cDNA from all RNA transcripts present in a sample. Each DNA fragment was end-labelled with biotin using terminal deoxynucleotidyl transferase (TdT) before being hybridized on a GeneChip hybridization Oven 640. Following hybridization and post-hybridization washes, the arrays were scanned using the Affymetrix GeneChip Scanner

3000 7G to generate the raw data (CEL file). The QC steps of the experiment were performed using Expression Console software (Affymetrix, Santa Clara, CA).

### Statistics

Statistical analysis of gene expression profiles was performed by using Partek Genomic Suite ver. 6.6 (Partek Inc., St. Louis, MO). Raw probe intensity values were background corrected by the Robust Multi-array Average (RMA) algorithm, quantile normalized and log transformed. Principal Component Analysis (PCA) was performed, as a quality control procedure, to verify that the division of the 21 individuals into N+ (lymph node involvement) and N- (without lymph node involvement) groups may be supported by a significant difference in gene expression profiles. Differentially expressed genes between 10 OSSC specimens of N- patients and 11 N+ patients were obtained by two-way ANOVA. Significant genes were those exhibiting at least 2-folds difference in gene expression between the two groups and FDR-corrected *p* values < 0.05.

### Gene set enrichment analysis

We have identified functional classes of differentially expressed genes or association with disease phenotypes by gene set enrichment analysis. We have compared the list of significant genes with the Gene Ontology FAT category of biological processes, molecular functions and cellular component, through DAVID ver. 6.8 [24, 25], WebGestalt (update 2013) [26] and GeneCodis3 [27]. Genes were first analyzed together, and then in subgroups made of 19 up and 22 down regulated genes only. DAVID was used to identify significant clusters of functional annotations of pathways and biological processes, while WebGestalt and GeneCodis3 were run against a number of Transcription Factor Binding Sites (TFBS) repositories with the aim to capture the putative regulatory sites.

### Expression data comparison

Comparison with public resources was carried out by using GENEVESTIGATOR 4.0 [28] and retrieving recalibrated expression data for published “oral mucosa” and “oral squamous cell carcinoma line” experiments (GEO accession numbers: GSE3526, GSE7307, GSE17913, GSE15101, GSE7307, GSE14905, GSE8056, GSE30999, GSE13355, GSE27280, GSE46239, GSE41664, GSE15101, GSE32924, GSE32473, GSE17539, GSE28914, GSE33169; GSE36133 for OSCC cell line SCC-4, SCC-9, SCC-15, SCC-25, BHY, BICR1 and HN).

### Functional network analysis

Functional networks of differentially expressed genes were inferred by Ingenuity Pathway Analysis (IPA spring 2017 release; QIAGEN, Redwood City, CA; www.qiagen.com/ingenuity). The entire inferential procedure worked on the Ingenuity Knowledge Base, which is a large structured collection of observations in various experimental contexts with nearly 6 million findings manually curated from the biomedical literature or integrated from third-party databases. Resulting networks were ranked according to their degree of relevance to the differentially expressed genes. The network scores are based on the hypergeometric distribution and were calculated with the right-tailed Fisher's Exact Test. Genes wired by the resulting networks were colored in green if up-regulated in N- (lymph node negative), red if up-regulated in N+ (lymph node positive) and white if not significantly deregulated into our datasets.
